# Recent Scientific Developments in Metastatic Prostate Cancer

**DOI:** 10.3390/cancers12113280

**Published:** 2020-11-06

**Authors:** Isabel Heidegger

**Affiliations:** Department of Urology, Medical University Innsbruck, 6020 Innsbruck, Austria; Isabel-maria.heidegger@i-med.ac.at

**Keywords:** metastatic hormone-sensitive prostate cancer, metastatic castration-resistant prostate cancer, personalized therapy, biomarker

In recent years, the treatment landscape of advanced prostate cancer has radically changed. While androgen deprivation monotherapy (ADT) was the standard of care for treating metastatic hormone-sensitive prostate cancer (mHSPC), in previous years, the addition of either docetaxel chemotherapy or second-generation ADTs (apalutamide, abiraterone acetate, and enzalutamide) to standard ADT demonstrated survival benefits, leading to ADT monotherapy no longer representing the standard for these patients. In addition, radiation therapy featured promising response rates in the subgroup of low metastatic burden defined as <4 bone metastases [1]. However, this rapid evolution is associated with crucial challenges in daily routine regarding the choice of treatment selection and the proper therapy sequencing, as there exist no prospective data to guide clinical decision after progression. Consequently, there is an urgent need for biomarkers that predict therapy response amongst others trough the introduction of novel high-throughput technologies or liquid biopsy on circulating cell-free nucleic acids that can be utilized in clinical management. Furthermore, better characterization of both the tumor itself and of the microenvironment is warranted in order to better understand the molecular alterations upon therapy pressure. 

Concerning metastatic castration-resistant prostate cancer (mCRPC), PARP inhibitors showed overall survival benefits in patients with BRCA1/2 mutations, leading to rucaparib and olaparib being approved by the FDA some months ago [2]. Furthermore, several clinical studies have shown the efficacy of 177Lu-labeled prostate-specific membrane antigen (PSMA) radioligand therapy for mCRPC; however, high-level evidence from randomized control trials is crucial for instituting this therapy in the routine clinical care of patients [3]. New frontiers in mCRPC treatment are combining therapies targeting different pathways, such as androgen receptor inhibitors together with PD-1 inhibitors or anti-CTLA4 antibodies; however, unraveling the mechanisms of synergy and resistance remains a challenge for the future [4]. Furthermore, integrative clinical sequencing analyses revealed that most mCRPCs harbor clinically actionable molecular alterations, thus, agents targeting the WNT or PI3K pathway as well as novel targets in cell cycle such as CDK4 inhibitors are currently tested in clinical trials [5].

In summary, regarding therapy of metastatic prostate cancer in 2020, we are moving into an era of personalized treatment, as discussed in this Special Issue.
